# What has functional connectivity and chemical neuroimaging in fibromyalgia taught us about the mechanisms and management of ‘centralized’ pain?

**DOI:** 10.1186/s13075-014-0425-0

**Published:** 2014-08-28

**Authors:** Vitaly Napadow, Richard E Harris

**Affiliations:** Athinoula A Martinos Center for Biomedical Imaging, Department of Radiology, Massachusetts General Hospital, Harvard Medical School, Charlestown, MA 02129 USA; Department of BioMedical Engineering, Kyunghee University, Yongin, 446-701 Korea; Chronic Pain and Fatigue Research Center, Department of Anesthesiology, University of Michigan, Ann Arbor, MI 48106 USA

## Abstract

Research suggests that fibromyalgia is a central, widespread pain syndrome supported by a generalized disturbance in central nervous system pain processing. Over the past decades, multiple lines of research have identified the locus for many functional, chronic pain disorders to the central nervous system, and the brain. In recent years, brain neuroimaging techniques have heralded a revolution in our understanding of chronic pain, as they have allowed researchers to non-invasively (or minimally invasively) evaluate human patients suffering from various pain disorders. While many neuroimaging techniques have been developed, growing interest in two specific imaging modalities has led to significant contributions to chronic pain research. For instance, resting functional connectivity magnetic resonance imaging (fcMRI) is a recent adaptation of fMRI that examines intrinsic brain connectivity - defined as synchronous oscillations of the fMRI signal that occurs in the resting basal state. Proton magnetic resonance spectroscopy (^1^H-MRS) is a non-invasive magnetic resonance imaging technique that can quantify the concentration of multiple metabolites within the human brain. This review will outline recent applications of the complementary imaging techniques - fcMRI and ^1^H-MRS - to improve our understanding of fibromyalgia pathophysiology and how pharmacological and non-pharmacological therapies contribute to analgesia in these patients. A better understanding of the brain in chronic pain, with specific linkage as to which neural processes relate to spontaneous pain perception and hyperalgesia, will greatly improve our ability to develop novel therapeutics. Neuroimaging will play a growing role in the translational research approaches needed to make this a reality.

## Fibromyalgia: a centralized pain disorder

Fibromyalgia (FM) is the second most common rheumatologic disorder, behind osteoarthritis, with 2 to 4% of the populations of industrialized countries affected [1]. Overall, it is estimated that FM costs American taxpayers over $20 billion a year in lost wages and disability [2]. In part, this burden on the US health care system stems from our lack of understanding of the specific pathophysiology of the disorder. Research suggests that FM is a central widespread pain syndrome [3,4]; however, it is uncertain if the observed neurobiological outcomes are causally related to development of this condition. That said, emerging data suggest a generalized disturbance in central nervous system pain processing, which leads individuals to sense pain throughout the body in the absence of inflammatory or patho-anatomic damage [5].

## Neuroimaging and pain: multiple windows into brain chemistry and function

Over the past decades, multiple lines of research have identified the locus for many functional, chronic pain disorders as the central nervous system, and the brain. Abnormal brain processing includes sensitization supported by aberrant inter-regional communication and other alterations in both structure and function, including neurotransmitter levels, all of which may ultimately maintain the chronic pain state. Brain neuroimaging techniques have heralded a revolution in our understanding of chronic pain, as they have allowed researchers to non-invasively (or minimally invasively) evaluate human patients suffering from various pain disorders.

Resting functional connectivity magnetic resonance imaging (fcMRI) is a recent adaptation of fMRI that examines intrinsic connectivity - defined as synchronous oscillations of the fMRI signal that occurs in the resting basal state. Intrinsic brain connectivity may be important for maintenance of synaptic connectivity and, as such, modulates the efficiency and extent of neuronal transmission between brain regions. Intrinsic connectivity, as measured by neuroimaging methods, follows known structural monosynaptic and polysynaptic pathways [6], likely reflecting meaningful neurophysiological activity [7] within known primary sensory, executive, and associative networks [8].

In simple terms, the subject is instructed to lie still inside the scanner and limit head motion. Analyses aim to understand patterns in the spontaneous fluctuations in the blood oxygenation level-dependent (BOLD) signal over time [9]. For instance, neural communication between distant brain regions is thought to be reflected by a significant correlation between fMRI signal time series from those regions. Thus, this technique is particularly sensitive to the investigation of brain networks, or co-activated assemblies of brain areas, and stable, reproducible networks processing both primary sensory and associative, and higher cognitive functions [10].

The spontaneous fluctuations in the resting fMRI signal demonstrate peak power at low frequencies (approximately 0.01 to 0.05 Hz). Thus, it is important to keep in mind that many of the correlations reported characterize neural fluctuations that occur over tens of seconds. Moreover, resting fcMRI analyses do not typically evaluate causal relationships between brain regions. Such analyses are suspect due to the fact that the hemodynamic response function (which converts neuronal activity to BOLD hemodynamic response) varies across the brain. Hence, preceding activation in one brain area compared to a second brain area may reflect true causal influence or, alternatively, a hemodynamic response function that peaks earlier in time compared to the second area [11].

Multiple techniques have been devised to evaluate functional brain connectivity. For correlational analyses, the main techniques are seed correlation and independent component analysis (ICA). For seed correlation, the fMRI signal is extracted from a seed region of interest, and is then correlated with the fMRI time series taken from all other brain voxels [12]. Alternatively, ICA is a data-driven technique that considers all voxels in the brain and clusters them into spatiotemporally distinct networks, which are spatially independent of one another [13].

Different fcMRI techniques have been applied to better understand brain processing related to pain. The application to chronic pain has been an important development, as clinical pain is much more difficult to study compared to evoked, or experimental, pain. Chronic pain manifests in spontaneous fluctuations of clinical pain. Such fluctuations shift connectivity between different brain regions and networks, a phenomenon that can be tracked with fcMRI.

A complementary neuroimaging technique to fcMRI is proton magnetic resonance spectroscopy (^1^H-MRS). This method is a non-invasive MRI technique that can quantify the concentration of multiple metabolites within the human brain. Of significance to the field of chronic pain, this method can be used to identify static as well as changing levels of specific brain neurotransmitters involved in pain processing and modulation. In this respect, ^1^H-MRS is complementary to fcMRI as it allows for the determination of the concentrations of specific neurotransmitters and brain metabolites involved in neural signaling that likely relate to the fcMRI signal. This technique generates a specific chemical spectrum, rather than a functional brain map, by exciting protons with characteristic resonance frequencies [14]. Once ^1^H-MRS spectra are acquired, they are analyzed offline to determine the relative concentrations of different metabolites [15]. This technique uses the same MRI scanner used to generate fcMRI data, and since ^1^H-MRS can be performed in the same scanning session, resting levels of brain metabolites and brain connectivity maps can be acquired with minimal time lag between acquisitions. Although the metabolites with the largest signal-to-noise ratios in ^1^H-MRS are N-acetyl-aspartate (NAA), choline (Cho), and creatine, recent investigations have targeted the brain’s main excitatory and inhibitory neurotransmitters - glutamate and gamma-amino-butyric acid (GABA), respectively. Emerging data suggest that these molecules may be key factors in how sensitized a given area of the cortex is to stimuli, and this in turn may be related to brain connectivity.

## ^1^H-MRS in fibromyalgia and other pain states

Alterations in the levels of multiple metabolites have been associated with various pain conditions thought to have underlying brain dysfunction. NAA is a metabolite that has historically been believed to be a marker of neuronal density and viability, and lower concentrations of this molecule are thought to be indicative of loss of neural function and activity [16,17]. Cho is also involved in neural activity as it plays a role in membrane integrity [16]. Creatine is a molecule that is involved in cellular energy and it plays a role in ATP metabolism and production. Since this molecule displays relatively stable levels across brain regions, it is sometimes used as a reference metabolite to which other molecules are normalized.

Over the past decade, the application of ^1^H-MRS methods to the investigation of chronic pain has gained popularity. Grachev and Apkarian [18] were the first to report lower NAA levels within the dorsolateral prefrontal cortex (DLPFC) of individuals with chronic low back pain compared with healthy controls. Subsequent reports of lower brain NAA levels have also been found in other chronic pain conditions, including neuropathic pain [19], trigeminal neuralgia [20], and headache/migraine [21,22]. This finding of lower NAA within the brain extends to patients with ‘centralized’ pain such as FM. Four independent trials have reported reductions in hippocampal NAA levels within individuals with FM [23-26]. In fact, Wood and colleagues [26] reported that reduced NAA within the hippocampus was also associated with greater symptom burden, as assessed by the Fibromyalgia Impact Questionnaire. These findings of lower NAA in the FM hippocampus are robust and likely reflect a true finding as a recent ^1^H-MRS meta-analysis of these same four trials, including 58 patients with FM and 38 pain free controls, showed a statistical reduction in hippocampal NAA in FM [27].

Although reduced NAA levels may be a common chemical alteration in chronic pain, there needs to be caution in the interpretation of this finding. The causal relationship between hippocampal NAA and pain has yet to be demonstrated. It is not known if lower NAA promotes the development of chronic pain, if chronic pain drives the reduction in NAA, or if both occur simultaneously. Lower hippocampal NAA may also simply be a marker for chronic pain and not be in the causal pathway. To resolve these questions, longitudinal studies that follow patients as they develop pain symptoms are needed.

Differences in other metabolite levels have also been found in centralized pain states. Our group found an association between Cho levels within the DLPFC and spontaneous clinical pain: greater Cho levels were positively correlated with self-reported chronic pain [28]. Emad and colleagues [25] reported elevations in Cho within the right hippocampus; however, the two trials by Fayed and colleagues [23,24] found reductions in Cho within the left hippocampus, while no differences in hippocampal Cho were found by Wood and colleagues [26].

While the molecular constituents of chronic centralized pain may involve NAA, and to some degree Cho, a model of how these metabolites play a role in pain presentation is lacking. In an effort to explore more traditional neural markers, recent work has begun to explore the role of brain glutamate and glutamine in centralized pain patients. As mentioned above, glutamate is the brain’s main excitatory neurotransmitter and it exerts its effects via binding to both ionotropic and metabotropic receptors. Ionotropic receptors are ligand-gated ion channels generally involved in fast synaptic transmission, which open up permeation pathways through the plasma membrane, allowing for fast changes in membrane potentials. Metabotropic receptors are G-protein-coupled receptors that typically signal through cytoplasmic second messengers and are more involved in modulation of neural activity. The role of glutamatergic neurotransmission in pain has been known for some time. For example, the development of neuropathic pain in preclinical models is thought to be, in part, a result of central sensitization, or central plasticity, involving both ionotropic as well as metabotropic glutamate receptors (reviewed in [29]). It remains to be seen if these processes are also involved in the brains of chronic pain patients that go on to develop centralized pain.

Our group was the first to use ^1^H-MRS to study glutamate and Glx (combined glutamate and glutamine) levels specifically in patients with chronic ‘centralized’ pain. In a longitudinal trial of acupuncture and sham acupuncture, we demonstrated that changes in Glx levels, specifically within the posterior insula cortex, tracked with changes in both experimental and clinical pain [30]; greater reductions in Glx were associated with greater improvements in both clinical and experimental pain. An important aspect of this study was that the changes in insular Glx were also associated with concomitant changes in the brain’s functional response to evoked pressure pain. This suggested the possibility that brain Glx levels may actually be associated with neural activity, and not simply a marker of pain. Similar findings have been found in other trials assessing the relationship between ^1^H-MRS-derived neurotransmitter levels and fMRI response [31]. Subsequently, our group compared glutamate and Glx levels within the posterior insula between FM patients and pain-free controls and found significantly elevated levels of these molecules in the FM patients. In both the FM and pain-free groups, however, the degree of Glx elevation was associated with evoked pain sensitivity, suggesting that glutamatergic activity in this region of the brain might be responsible, in part, for the ‘gain setting’ on central neural pain processing [32].

Findings of elevated Glx within the FM brain have also been reported by other groups, albeit focusing on different brain regions. Elevations in the levels of Glx have been reported in the amygdala [33], the posterior cingulate [34], and the ventral lateral prefrontal cortex [35] of individuals with FM. The emerging view is that there may be multiple loci within the FM brain wherein elevated Glx may play a role in chronic pain symptoms. This is in agreement with the fact that many of these patients complain of symptoms, in addition to pain, that also have brain-based neurobiological underpinnings, namely mood disturbance, poor sleep, cognitive dysfunction, and fatigue. However, there does not appear to be global or ‘non-specific’ elevations in brain Glx as these findings have not been detected in every region examined. No elevations have been detected in the anterior insula [32] or the prefrontal cortex [33].

These findings could represent more glutamate within synaptic vesicles, higher numbers or densities of glutamatergic synapses, or even less reuptake of glutamate from the synaptic cleft in centralized pain; all of which could enhance excitatory neurotransmission and subsequent pain. In neuropathic pain, plastic changes occurring in the spinal cord and brain are thought to result from some of these mechanisms. These changes in brain Glx in centralized pain may also reflect processes similar to central sensitization that have been reported in animal models [29] and have been proposed in FM [36].

However, some limitations need to be acknowledged before we can make these conclusions. First, ^1^H-MRS-derived levels of glutamate are not purely estimates of glutamate. As mentioned above, glutamine has magnetic resonances that overlap glutamate [14], thus precluding the ability to assign altered levels solely to glutamate. Second, glutamate is not solely a neurotransmitter. Within the brain, glutamate is involved in the citric acid cycle and synthesis of ATP, glutamate levels thus reflecting not only neurotransmission but also metabolism. Finally, ^1^H-MRS voxels contain multiple cell types. The Glx levels detected with ^1^H-MRS are present not only in the neurons themselves, but also glia and other cell types within the brain. The cellular compartment from where the elevated glutamate signal originates in FM is not known, and changes in glutamate and Glx observed in FM may be at regions remote from the synapse.

While interpretation of ^1^H-MRS levels of glutamate are somewhat problematic, the assessment of the brain’s major inhibitory neurotransmitter, GABA, may be easier. Unlike glutamate and Glx, GABA does not play a role in metabolism and, as such, ^1^H-MRS-derived GABA levels may be more indicative of neural activity. Interestingly, recent results suggest that decreased fMRI BOLD signals are associated with higher GABA levels [37], although other processes may be operative as the fMRI BOLD signal involves non-neuronal factors (that is, the hemodynamic response).

Similar to glutamate, GABA binds to both ionotropic as well as metabotropic receptors; however, unlike glutamate the binding of GABA typically leads to neuronal inhibition through the opening of an electric shunt with the neuronal membrane potential. GABA receptors are widely distributed throughout the brain and spinal cord where they are thought to modulate pain processing. The first studies showing that GABA plays a critical role in pain transmission involved demonstrating that baclofen, a GABA-B receptor agonist, blocked pain in preclinical models of acute and chronic pain [38]. These effects were likely mediated by both spinal and supraspinal GABA-B receptors. Interestingly, decreases in insular GABA levels exacerbate pain whereas blocking GABA degradation, within this structure, relieves pain [39]. These results suggest that GABA may play a role in the pathophysiology of some chronic pain states.

We were the first to report that GABA levels are altered within the centralized pain brain [40]. Although this was a pilot study, within a sample of 16 FM patients and 17 age- and sex-matched healthy controls, we found lower GABA levels specifically within the anterior insula. No reductions were detected within the posterior insula; however, lower GABA levels within the posterior insula were associated with greater sensitivity to experimental pain. These findings suggested that lower insular GABA may also play a role in pain, namely neuronal disinhibition. Our results also raise the intriguing possibility of a neurotransmitter imbalance within the insula of FM patients. There may be an elevation in the ratio of insular Glx/GABA (excitatory/inhibitory) in centralized pain.

## fcMRI in fibromyalgia

fcMRI is a technique for analysis of the resting state BOLD time series; that is, when no experimental task is imposed on the subject. Studies applying fcMRI techniques to FM complement evoked pain fMRI studies, as the latter mainly interrogate hyperalgesia and allodynia phenomena in FM patients, but do not evaluate the brain correlates of spontaneous clinical pain in these patients. Napadow and colleagues evaluated resting, or intrinsic, brain connectivity in FM patients using ICA [41]. They found altered connectivity between the insula and the default mode network (DMN) and the executive attention network (EAN; also known as the frontoparietal control network). The DMN [8,9] is a constellation of brain regions thought to be engaged in self-referential cognition, which are ‘deactivated’ during various externally focused task conditions. The DMN includes the inferior parietal lobule, the posterior cingulate cortex (PCC) and precuneus, medial prefrontal cortex (mPFC), the hippocampal formation, and the lateral temporal cortex [42]. Pain is known to influence both DMN response and cognitive capacity. While acute experimental pain induces DMN deactivation in healthy subjects [43], chronic back pain is associated with mitigated DMN deactivation to visual attention tasks [44]. The EAN comprises the dorsolateral prefrontal and posterior parietal cortices and is involved in executive control over behavior. The DMN showed greater connectivity to the insula cortex and secondary somatosensory cortex (S2) (brain regions known to process evoked experimental pain and somatosensation), while the EAN showed greater intra-network connectivity in FM patients. The medial visual network, which was used as a control, did not show functional differences between patients and controls. Moreover, both the DMN and EAN were more connected to the insula in patients reporting greater spontaneous clinical pain at the time of the scan. This suggested a close link between DMN-insula connectivity and clinical pain.

Reduced resting connectivity within the somatosensory system and increased connectivity between the DMN and somatosensory processing regions such as S2 (as also mentioned above) were recently reported by Pujol and colleagues [45]. Such independent, confirmatory data are important for any neuroimaging-based markers of disease in FM, and further research is needed. Interestingly, this study also found altered connectivity with brain regions supporting visual and auditory processing, which may relate to the multi-sensory dysfunction sometimes reported in these patients.

In a different study, Cifre and colleagues [46] used a seed voxel region of interest approach and showed a pattern of both increased and decreased brain connectivity in FM patients. Increased connectivity was found between DMN areas such as mPFC and PCC and also between anterior cingulate cortex and the insula. These results support the fact that DMN and insula resting connectivity is disrupted in FM. Some results may also extend to other pain conditions, as Kucyi and colleagues [47] also found increased mPFC to PCC connectivity in patients with temporomandibular disorder, with greater mPFC-PCC connectivity associated with greater rumination about pain reported by the patients.

Ceko and colleagues [48] explored structural and fMRI changes in FM patients, and found an interesting association with age. Younger, but not older, FM patients showed decoupling between the insula and anterior mid-cingulate cortex, two brain regions that are normally strongly connected in healthy adults, as part of a salience network.

In addition to altered connectivity, potential spectral power differences have also been explored in resting fcMRI data. Kim and colleagues [49] reported increased frequency power (for a broad 0.01 to 0.25 Hz band) in somatosensory (primary somatosensory cortex, S1), cognitive (DLPFC) and affective (amygdala) brain regions in FM patients.

## Relationship between the functional and chemical imaging findings and pain

It is becoming increasingly apparent that altered connectivity and neurochemistry are present within the FM brain. However, it is currently unknown if these processes are operative in the same brain regions and within the same individuals. No group to date has explored the relationship between ^1^H-MRS-derived neurotransmitter levels and functional connectivity in a chronic pain cohort. Recent work has investigated the relationship between Glx- and GABA-derived spectroscopy values within the posterior cingulate and connectivity of this structure to the rest of the DMN [50]. The authors find that individuals with greater concentrations of Glx and lower concentrations of GABA within the posterior cingulate have stronger connectivity values with other DMN regions. One approach that may be particularly informative in FM would be to explore the association between insula connectivity and Glx/GABA levels in the same patient cohort. For instance, a connectivity seed voxel could be placed within the insula that matches the ^1^H-MRS voxel in position and shape, and analyses could determine if Glx within the insula is related to connectivity of this structure with the rest of the brain (see below). This type of multi-modal imaging would be particularly informative and may provide synergistic insights into central neurobiological pathways that are dysregulated in chronic pain. Alternatively, Glx and GABA concentration in the insula may also influence functional connectivity between other brain regions and networks, as the insula has widely distributed excitatory and inhibitory connections throughout the brain.

## Neuroimaging of treatment effects

Neuroimaging techniques have been applied to further our understanding of the brain mechanisms supporting pharmacological and non-pharmacological analgesic therapies for FM. For instance, Napadow and colleagues [51] demonstrated that DMN-insula connectivity, which was increased in FM patients, was reduced following 4 weeks of non-pharmacological acupuncture and sham acupuncture therapy, which reduced pain in these patients. The authors suggested that connectivity between the DMN and insula may serve as a possible surrogate biomarker for pain reduction in FM.

Recently, pregabalin, a pharmacologic intervention approved by the United States Food and Drug Administration for the treatment of FM, has been investigated in a multi-modal MRI study [52]. Consistent with the preclinical mechanism of action of this compound (that is, a reduction in the release of glutamate into the synapse), Harris and Napadow and colleagues found that pregabalin reduced Glx levels in the posterior insula [52]. Moreover higher pre-treatment levels of Glx were associated with greater subsequent reductions in sensitivity to experimental pressure pain. Patients that had greater reductions in clinical pain also displayed greater concomitant reductions in functional connectivity between the posterior insula and DMN structures, consistent with earlier reports linking DMN-insula connectivity with spontaneous fluctuations in clinical pain.

## Where future studies are needed

While studies to date have made tremendous progress in delineating the brain mechanisms supporting persistent pain in FM patients, future studies will need to better link neuroplastic change in the brain with both pain and non-pain clinically relevant outcomes. As FM is a multi-dimensional disorder, non-pain outcomes (such as fatigue, cognitive deficits, mood disturbance, and poor sleep) should also be explored with brain imaging approaches. A comprehensive mechanistic model that involves altered central nervous system physiology is much needed to understand how different symptoms co-occur in FM patients. Future studies will need to accurately phenotype FM patients to determine the relative levels of these comorbid symptoms in order to ascertain which brain outcomes are related to specific clinical outcomes. Given the emerging belief that FM may be an umbrella diagnosis for multiple different subtypes of patients suffering from whole-body pain, differences in brain alterations between different patient subgroups may help explain symptom heterogeneity.

Finally, several potential confounds inherent to the previously described neuroimaging markers need to be mentioned. Firstly, cardiorespiratory artifacts and subject motion inside the MRI scanner can significantly affect the neuroimaging markers noted above. For instance, head motion has been shown to reduce spectral power at low frequencies and increase power at high frequencies (likely due to the jerky nature of such motion). Importantly, these motion-related effects are greater in association networks such as the DMN and fronto-parietal control network [53]. As patients tend to move more than healthy control subjects, these effects need to be disentangled from real neuroplastic changes. At the least, head motion should be quantified and reported. Furthermore, physiological monitoring should be used in resting connectivity analyses, in order to remove, or mitigate, cardiorespiratory artifacts in the data. From a clinical perspective, many FM patients take medications that could alter brain outcomes, potentially making it difficult to assign altered brain outcomes to the presence of the disorder as opposed to the confounding effects of certain drugs. Finally, most neuroimaging studies are performed on relatively small sample sizes; for example, it is not uncommon for a neuroimaging trial to enroll fewer than 30 patients. While small sample sizes may still provide sufficient power for neuroimaging outcomes, it does limit our ability to generalize any one finding into the larger population of centralized pain patients. One way around this problem is the generation of shared data across institutions as in the large NIH-funded Multidisciplinary Approach to the Study of Chronic Pelvic Pain (MAPP) initiative, which is focused on the characterization of patients suffering from pelvic pain. In the future, these types of ‘big data’ approaches will be needed to help us better understand centralized pain disorders and other related syndromes.

## Conclusion

fcMRI and ^1^H-MRS analyses have identified significant alterations in brain function and neurotransmitter concentration in the FM brain. While these analysis approaches are relatively new and still evolving, future studies with greater power will better link these brain changes with clinically relevant outcome metrics. Such research will lead to an improved understanding of how brain changes reflect and even maintain persistent pain in FM.

## Note

This article is part of the series on *New perspectives in fibromyalgia*, edited by Daniel Clauw. Other articles in this series can be found at http://arthritis-research.com/series/fibromyalgia

## Abbreviations

^1^H-MRS: Proton magnetic resonance spectroscopy
BOLD: Blood oxygenation level-dependent
Cho: Choline
DLPFC: Dorsolateral prefrontal cortex
DMN: Default mode network
EAN: Executive attention network
fcMRI: functional connectivity magnetic resonance imaging
FM: Fibromyalgia
fMRI: functional magnetic resonance imaging
GABA: Gamma-amino-butyric acid
Glx: Glutamate plus glutamine
ICA: Independent component analysis
mPFC: medial prefrontal cortex
NAA: N-acetyl acetate
PCC: Posterior cingulate cortex
S2: Secondary somatosensory cortex
